# Foraging at wastewater treatment works affects brown adipose tissue fatty acid profiles in banana bats

**DOI:** 10.1242/bio.013524

**Published:** 2016-01-06

**Authors:** Kate Hill, Sunet van Aswegen, M. Corrie Schoeman, Sarina Claassens, Peet Jansen van Rensburg, Samantha Naidoo, Dalene Vosloo

**Affiliations:** 1 School of Life Sciences, University of KwaZulu-Natal: Westville Campus, Private Bag X54001, Durban 4000, South Africa; 2 Unit for Environmental Sciences and Management, North-West University: Potchefstroom Campus, Private Bag X6001, Potchefstroom 2520, South Africa; 3 Human Metabolomics, North-West University: Potchefstroom Campus, Private Bag X6001, Potchefstroom 2520, South Africa

**Keywords:** Banana bats, Fatty acid profile, Wastewater treatment works, Chironomid midges

## Abstract

In this study we tested the hypothesis that the decrease in habitat quality at wastewater treatment works (WWTW), such as limited prey diversity and exposure to the toxic cocktail of pollutants, affect fatty acid profiles of interscapular brown adipose tissue (iBrAT) in bats. Further, the antioxidant capacity of oxidative tissues such as pectoral and cardiac muscle may not be adequate to protect those tissues against reactive molecules resulting from polyunsaturated fatty acid auto-oxidation in the WWTW bats. Bats were sampled at two urban WWTW, and two unpolluted reference sites in KwaZulu-Natal, South Africa. Brown adipose tissue (BrAT) mass was lower in WWTW bats than in reference site bats. We found lower levels of saturated phospholipid fatty acids and higher levels of mono- and polyunsaturated fatty acids in WWTW bats than in reference site bats, while C18 desaturation and n-6 to n-3 ratios were higher in the WWTW bats. This was not associated with high lipid peroxidation levels in pectoral and cardiac muscle. Combined, these results indicate that WWTW bats rely on iBrAT as an energy source, and opportunistic foraging on abundant, pollutant-tolerant prey may change fatty acid profiles in their tissue, with possible effects on mitochondrial functioning, torpor and energy usage.

## Abstract (precis)

**Summary:** Brown adipose tissue of banana bats foraging at wastewater treatment works contained lower saturated and higher mono- and polyunsaturated phospholipid fatty acids than that of bats foraging at reference sites.

## INTRODUCTION

Bats (Order Chiroptera) have exceptional lifespans for their size (Barclay and Harder, 2003), which challenges both the widely accepted positive association between size and lifespan (Seim et al., 2013), and the oxidative theory of ageing (Munshi-South and Wilkinson, 2010). This has been attributed to a combination of factors including high levels of tissue antioxidants (Wilhelm Filho et al., 2007), low levels of pro-oxidants (Brunet-Rossinni, 2004), slow reproduction output (Wilkinson and South, 2002), low predation rate and their ability to enter hibernation (Turbill et al., 2012; Wilkinson and South, 2002) or torpor (Turbill et al., 2012). These traits indicate that bats have huge potential as bioindicators (Jones et al., 2009), yet they also render bats vulnerable to environmental stresses, many of which are induced by humans. Bat populations appear to be declining almost everywhere in the world, and even species usually considered abundant have experienced declines (Stebbings, 1988). Two key classes of environmental stresses include climate change and natural habitat conversion into urban and agricultural landscapes, and there is evidence that these stresses affect reproductive output (Adams, 2010; Estrada and Coates-Estrada, 2002), predation rate (Presley et al., 2009) and hibernation (Boyles and Willis, 2009). However, studies on the impact of some these environmental stressors on bat physiology are scarce.

One environmental feature that poses potential risk to wildlife is wastewater treatment works (WWTW), where water received from domestic and industrial areas are treated, and purified effluent is usually released into natural water bodies. WWTW sludge is known to contain a cocktail of pollutants such as metals, disinfectants, pharmaceuticals, and steroids (Kinney et al., 2006; Spiegel et al., 1985). The open sludge tanks of WWTW provide an excellent breeding ground for pollutant tolerant insects such as chironomid midges which, in turn, are utilised by predators such as birds and bats (Park and Cristinacce, 2006). Recent studies have shown that the benefits of this abundant food source may carry physical, haematological, immunological, and molecular costs to the predators (Naidoo et al., 2013, 2015; Pilosof et al., 2014). However, how foraging at WWTW affects brown adipose tissue fatty acid profiles is not known. A predominantly chironomid diet may affect fatty acid intake as chironomids have higher levels of linoleic acid (Schalk and Brigham, 1995), but lower total polyunsaturated fatty acid (PUFA) levels (Ghioni et al., 1996) than nocturnal insects such as lepidopterans that may predominate in the diets of bats at sites not impacted by WWTW. Moreover, adiposity and fatty acid profiles may change through exposure to certain toxicants contained within the wastewater (Decherf and Demeneix, 2011), and artificial light, such as that found at well-lit WWTW, may affect adiposity in mammals through attenuation of brown adipose tissue (BrAT) activity (Kooijman et al., 2015).

BrAT functions as a storage organ (Chalvardjian, 1964) and regulates non-shivering thermogenesis. It is among others found in the interscapular region of small mammals (Cannon and Nedergaard, 2004), and its dark colour stems from rich vascularisation and densely packed mitochondria (Jennissen et al., 2011). Mitochondrial function can be affected by its phospholipid fatty acid profiles, which in turn can be influenced by dietary fatty acids (Chalvardjian, 1964). As with other mammals, bats need fatty acids as an energy source. Saturated and long chain fatty acids are energy dense whereas shorter, unsaturated fatty acids are easy to metabolise and thus essential in providing energy in harsh conditions, such as during migration, hibernation or food shortages (Chalvardjian, 1964; Levin et al., 2013; McGuire et al., 2013). Furthermore, mammals can endogenously produce certain fatty acids through elongation and desaturation. For example, C18:0, a saturated fatty acid (SFA) is produced from C16:0 by elongation and it can then be desaturated to C18:1n-9, a mono-unsaturated fatty acid (MUFA) (Guillou et al., 2010). Mammals can produce unsaturated fatty acids in the n-9 series through desaturation and/or elongation, but lack the enzymes needed to endogenously produce fatty acids in the n-3 and n-6 series. These essential fatty acids (EFAs) need to be obtained from food, or produced from food-derived precursors (Calder, 2006) and animals, including bats, select foods high in EFAs if needed (Levin et al., 2013; Schalk and Brigham, 1995).

Abbreviations:

WWTW: wastewater treatment works
*N. nana*: *Neoromicia nana*
TAC: total antioxidant capacity
ATP: adenosine triphosphate
ROS: reactive oxygen species
SFA: saturated fatty acid
PUFA: polyunsaturated fatty acid
MUFA: monounsaturated fatty acid
EFAs: essential fatty acids
BrAT: brown adipose tissue
iBrAT: interscapular brown adipose tissue
NST: non-shivering thermogenesis
*ucp*-1: uncoupling protein-1 gene
HNE: aldehyde 4-hydroxy-2-nonenal
TBT: tributyl tin
TBARS: thiobarbituric acid-reactive substances
NL: neutral lipid
PL: phospholipid
FAMEs: fatty acid methyl esters
TMCS: trimethylchlorosilane
BSTFA: N,O-bis-trimethylsilyl-trifluoroacetamide
TCEP: tris(2-carboxyethyl) phosphine
t-BuOOH: *tert*-Butyl hyderperoxide
ANOVA: analysis of variance
NMDS: non-metric multidimensional scaling.

BrAT phospholipids can also be selectively mobilised to provide much-needed fatty acids to other tissues, such as highly oxidative muscles (Groscolas and Herzberg, 1997). EFAs, such as PUFAs, are important in maintaining torpor by allowing minimum body temperature to decrease without the risk of death, and maintaining basal metabolic rates during periods of inactivity (Chalvardjian, 1964; Ocloo, 2006). In fact, an optimal n-6 to n-3 PUFA ratio in tissues is even more important than merely high EFA concentrations (Ruf and Arnold, 2008). There is a trade-off between the benefit of high n-6 to n-3 ratios to maintain functionality in torpor, and the potentially damaging oxidation products of n-3 PUFAs, such as aldehyde 4-hydroxy-2-nonenal (HNE) Animals will most likely not maintain high n-6 to n-3 PUFA ratios if they do not frequent torpor. Damaging effects of HNE is not restricted only to the tissue in which it is produced, but it can affect other target tissues, for example cardiac muscle is vulnerable to the effects of HNE (Ruf and Arnold, 2008). Although more unsaturated fatty acids are important to maintain function, high C16 and C18 desaturation indices (MUFA:SFA) in BrAT fatty acids are considered adequate markers for diabetes and obesity in rats (Jeyakumar et al., 2009).

*Neoromicia nana* (the banana bat; Family Vespertilionidae) is an urban adapter (*sensu* Jung and Kalko, 2011) that opportunistically feeds on chironomid midges emerging from tank sites at WWTW and riverine habitats downstream from WWTW (Naidoo et al., 2013). However, heavy metals in the water at WWTW accumulate in *N. nana* liver, kidney and muscle tissues (Naidoo et al., 2013) with sub-lethal haemotological and genotoxic consequences (Naidoo et al., 2015). Additionally, the detoxification organs of *N. nana* at WWTW differ significantly from those of bats at reference sites: increased essential metal (Fe, Zn, and Cu) and one mineral nutrient (K) content; more histopathological lesions; and increased organ size (Naidoo et al., 2016). In this study we tested the hypothesis that the decrease in habitat quality at WWTW (limited prey diversity, exposure to the toxic cocktail of pollutants and high illumination at night) affect fatty acid profiles in thermogenic, interscapular BrAT (iBrAT) of *N. nana*. Specifically, we predicted that iBrAT from bats sampled at WWTW contain lower SFAs and higher PUFAs, especially linoleic acid, than from bats at reference sites, resulting in unfavourable n-6 to n-3 ratios and high desaturation indices. We also predicted that the antioxidant capacity in WWTW bats are not sufficient to protect other oxidative tissues such as cardiac and flight muscles, from end products associated with n-3 auto-oxidation. We therefore expected an increase in lipid hydroperoxide levels if the antioxidant defence is overwhelmed in WWTW bats.

## RESULTS

### Morphometrics

**Fig. 1.**
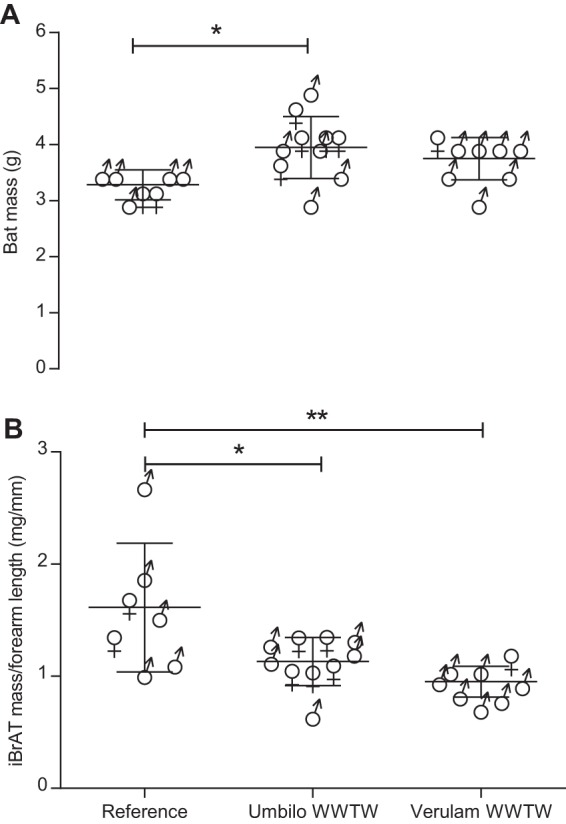
**Bat mass and interscapular brown adipose tissue (iBrAT) mass scaled to forearm length in *N. nana*.** (A) Bat mass and (B) iBrAT mass of animals collected from reference sites Verulam WWTW and Umbilo WWTW, compared with reference animals. Asterisks indicate results of a Kruskal–Wallis test in A and a one-way ANOVA followed by Tukey HSD test in B. *P<0.05; **P<0.01. Data represented as mean±s.d.

Bats from all sites were similar in size as indicated by forearm length measurements (*P*>0.05; *F*_2,22_=0.44) and adults. Body mass of bats was greater at Umbilo WWTW than at the reference sites (*P*<0.05; KW statistic=8.29), but iBrAT mass was significantly lower at both WWTW sites than the reference sites (*F*_2,22_=7.55, *P*<0.05) (Fig. 1A). Overall, there was a significantly negative relationship between iBrAT mass and body mass (*r*=−0.42; *P*<0.05).

### Lipid profile

**Fig. 2.**
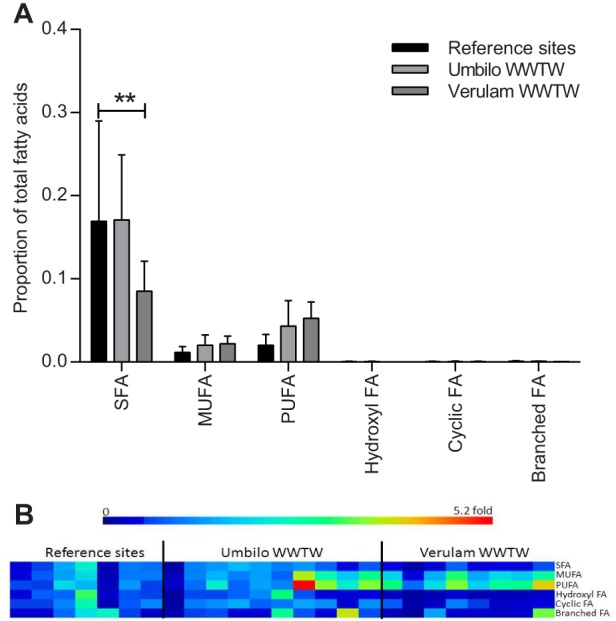
**Phospholipid fatty acids per class in *N. nana* brown adipose tissue.** (A) Mean phospholipid fatty acid concentrations (error bars=s.d.) per class in brown adipose tissue of *N. nana* bats collected from reference sites, Verulam WWTW and Umbilo WWTW. Asterisks indicate significant differences between sites as derived from two-way ANOVA followed by Tukey's multiple comparisons test. **P<0.01. (B) Heat map representing fatty acid concentrations in brown adipose tissue of individual bats relative to the average of the reference site, with each column presenting an individual bat.

In iBrAT, phospholipid fatty acids comprised 20.3% of all fatty acids at reference sites, 23.6% at Umbilo WWTW and 16.0% at Verulam WWTW, but there was no significant difference between unpolluted and polluted sites. Fatty acid classes identified from iBrAT phospholipids differed among classes (*P*<0.0001, *F*_5,132_=59.45) and not between sites (*P*>0.05,*F*_2,132_=1.55), and the interaction between class and site was significant (*P*<0.05, *F*_10,132_=3.21). Posthoc Tukey tests revealed that SFAs were the most abundant of all fatty acid classes at all sites (all *P*s<0.05), except for Verulam WWTW, where there was no difference between SFA and PUFA (Fig. 2A).

Although averages were not significant, the heat map (Fig. 2B) showed that all bats from Verulam and some from Umbilo WWTW had much higher (up to 16-fold) MUFA and PUFA levels than most bats from the reference sites.

**Fig. 3.**
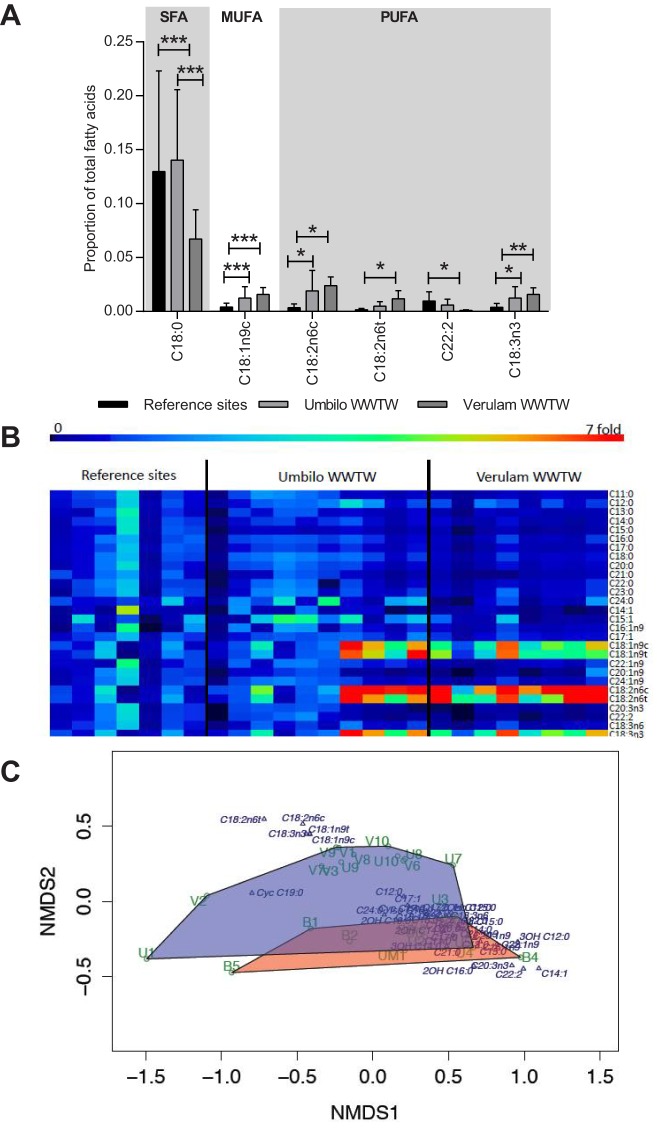
**Phospholipid fatty acids in *N. nana* brown adipose tissue.** (A) Mean BAT phospholipid fatty acid concentrations (error bars=s.d.) in brown adipose tissue of *N. nana* bats collected from reference sites, Verulam WWTW and Umbilo WWTW. Asterisks indicate significant differences between sites as derived from two-way ANOVA followed by Tukey's multiple comparisons test. *P<0.05; **P<0.01; ***P<0.001. Only fatty acids that were significantly different between sites are shown. (B) Heat map representing fatty acid concentrations in brown adipose tissue of individual bats relative to the average of the reference site, with each column presenting an individual bat. (C) Non-metric multidimensional scaling of fatty acids of bats. Convex hulls connect bats from polluted sites [U (Umbilo WWTW) and V (Verulam WWTW)] and unpolluted sites [UM (Umdoni) and B (Buffelsdrift farm)]. The blue shaded area represents WWTW bats, and the orange shaded area represents reference site bats.

Specific SFAs differed within sites (*P*<0.0001, *F*_2,286_=67.97) and between sites (*P*<0.05, *F*_2,286_=4.318), and the interaction between fatty acid profiles within and between and sites was significant (*P*<0.0001, *F*_24,286_=2.802). Posthoc Tukey tests confirmed that C18:0 (stearic acid) was the most abundant SFA in bats from all sites, followed by C16:0 (palmitic acid). C18:0 was higher in reference site bats than in Verulam WWTW bats, and also differed between WWTW (*P*<0.0001, Fig. 3A). C18:1n9c (oleic acid) was the most abundant MUFA, and was significantly higher in Verulam WWTW bats than in reference site bats, (overall *P*<0.05, sites: *F*_2,198_=3.107; fatty acid: *F*_8,198_=33.04; interaction *F*_16,198_=4.203; Fig. 3A).

PUFAs were significantly different between sites (*P*<0.001, *F*_2,132_=77.45) and within sites (*P*<0.001, *F*_5,132_=17.21), and the interaction between and within sites was also significant (*P*<0.001, *F*_10,132_=5.022). C22:2 (docosadienoic acid) was the most abundant PUFA in reference site bats, and significantly higher than in Verulam WWTW bats (*P*<0.05), while C18:2n6c (linoleic acid) was the most abundant PUFA in bats from Verulam and Umbilo WWTW, followed by C18:3n3 (alpha linolenic acid). Both PUFAs were significantly higher in WWTW bats than in reference site bats (Tukey test *P*<0.05, Fig. 3A), while C18:2n6t was also significantly higher in Verulam WWTW bats than in reference site bats (*P*<0.05).

The heat map of specific fatty acids reflects the high inter-individual variation, although those fatty acids that were significantly higher at especially the Verulam WWTW (oleic acid, elaidic acid, linoleic acid, linolelaidic acid and alpha linolenic acid) were high in all individuals from this site (Fig. 3B).

NMDS (final stress=0.037, linear fit of ordination distance and observed dissimilarity *R*^2^=0.99) separated the fatty acid composition of insectivorous bats into polluted and unpolluted sites with some overlap (Fig. 3C). Five out of six PUFAs (C18:2n6c, C18:2n6t, C18:3n3, C20:3n3 and C22:2) and four out of nine monounsaturated fatty acids (C18:1n9c, C18:1n9t, C22:1n9 and C14:1), as well as two hydroxyl fatty acids (2OH C16:0, 3OH C12:0) grouped outside the convex hulls connecting the polluted and unpolluted sites.

**Fig. 4.**
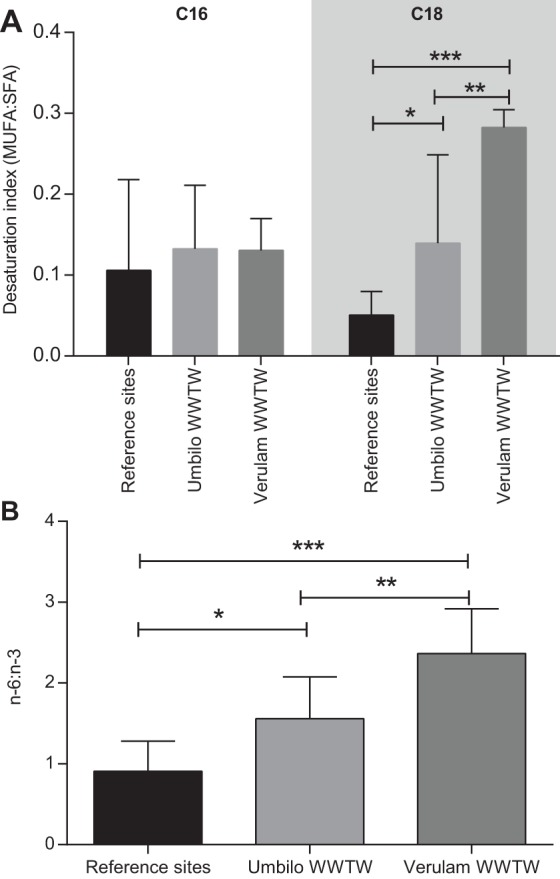
**N-6:n-3 ratio and C18, but not C16 desaturation index is increased in iBrAT phospholipids of WWTW bats.** (A) C16 and C18 desaturation indices and (B) n-6:n-3 ratio of animals collected from reference sites Verulam WWTW and Umbilo WWTW, compared with reference animals. Asterisks indicate significant differences between WWTW and reference sites as determined with Kruskal–Wallis statistics and Dunn's multiple comparisons test for A and a one-way ANOVA followed by Tukey's multiple comparisons test for B. *P<0.05; **P<0.01; ***P<0.001). Data represented as mean±s.d.

The n-6 to n-3 PUFA ratios were significantly higher in Verulam bats than in reference site bats (*P*<0.0001, *F*_2,22_=16.42; Fig. 4A). The C18 desaturation index was significantly higher in bats from Verulam WWTW than in reference site bats (*P*<0.01, KW statistic 15.14), whereas there were no differences in the C16 desaturation index between sites (Fig. 4B).

### Total antioxidant capacity

**Fig. 5.**
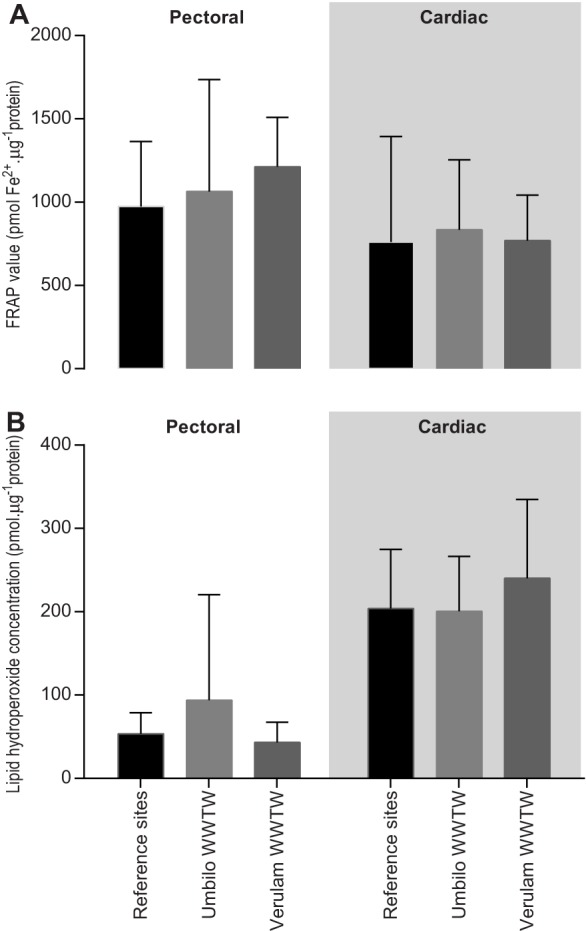
**Total antioxidant capacity and lipid peroxidation vary between tissue types, but not between collection site in *N. nana.*** (A) Total antioxidant capacity (TAC) and (B) lipid hydroperoxides per heart and pectoral muscle protein concentration in animals collected from reference sites Verulam WWTW and Umbilo WWTW, compared with reference animals. Kruskal–Wallis statistics and Dunn's multiple comparisons test. Responses at the different sites did not differ significantly. Data represented as mean±s.d.

Total antioxidant capacity (TAC) represented by pmoles of Fe^2+^ per µg of total protein in *N. nana* differed between tissue type (*P*<0.05, *F*_1,46_=4.963), but not between sites (*P*>0.05) (Fig. 5A).

### Lipid peroxidation

There were no significant differences between lipid hydroperoxides (pmole µg^−1^ protein) in the heart muscle tissue types across sites or the pectoral muscle tissue across sites (*P*>0.05). Lipid peroxidation in cardiac muscle was consistently higher than in pectoral muscle (*P*>0.001; *F*_1,27_=29.27). No correlation was found between antioxidant capacity and lipid peroxidation values in either tissue type, nor between the iBrAT n-6 to n-3 PUFA ratios and cardiac and pectoral lipid peroxidation (*P*>0.05) (Fig. 5B).

## DISCUSSION

We found support for the hypothesis that phospholipid fatty acid profiles in thermogenic iBrAT differ between WWTW bats and bats foraging at sites not affected by WWTW. In accordance with our predictions only two SFAs were found among the five most abundant fatty acids of bats at the Umbilo and Verulam WWTW, together with one MUFA and two PUFAs [Umbilo WWTW: stearic acid (SFA)>palmitic acid (SFA)>linoleic acid (PUFA)>oleic acid (MUFA)>linolenic acid; Verulam WWTW: stearic acid (SFA)>linoleic acid (PUFA)>oleic acid (MUFA)>linolenic acid (PUFA)>palmitic acid (SFA)]. By contrast, the five most abundant fatty acids in bats at the reference sites included three SFAs, One MUFA and one PUFA [stearic acid (SFA)>palmitic acid (SFA)>docosadienoic acid (PUFA)>heptadecanoic acid (SFA)>oleic acid (MUFA)]. Bats that experience caloric restriction, or exposure to stressful conditions, may fuel metabolism with shorter, less saturated fatty acids because these fatty acids allow for easier oxidation (McGuire et al., 2013). For example, there is evidence of subtle changes in saturation of muscle phospholipids of migrating bats (McGuire et al., 2013), and BrAT in starving rats (Chalvardjian, 1964). Mammals utilise dietary PUFAs to maintain function during torpor and hibernation. Phospholipid PUFAs are not only important for allowing membrane fluidity at lower body temperatures, but to also maintain mitochondrial efficiency and whole animal energy saving during these inactive periods. In ground squirrels, this was only achieved when fed a diet rich in linoleic acid (Gerson et al., 2008). Given that chironomid midges are high in linoleic acid (Schalk and Brigham, 1995), the opportunistic feeding on dipterans at WWTW by *N. nana* (Naidoo et al., 2013), may provide the bats with the fatty acids needed to maintain function in this polluted environment, as evident from the high phospholipid PUFA levels in the iBrAT of WWTW bats. Because fuelling stress responses are metabolically costly (Hochachka and Somero, 2002), and there is evidence that the physiology of *N nana* at WWTW is impacted by these polluted environments (Naidoo et al., 2015), one would expect mitochondrial efficiency to be of great importance to these bats.

Our results show that iBrAT of WWTW bats, especially at Verulam contain higher n-6 to n-3 PUFA ratios than bats at reference sites. High muscle phospholipid n-6 to n-3 PUFA ratios are associated with torpor use in mammals (McGuire et al., 2013; Ruf and Arnold, 2008), while dietary n-3 fatty acids are directly associated with the activation of non-shivering thermogenesis in BrAT during arousal from torpor via uncoupling protein-1 (Takahashi and Ide, 2000). These results suggest that the Verulam WWTW bats have a higher need for, and capacity to utilise torpor, yet whether these bats indeed utilise deeper or longer torpor bouts in the wild, still needs to be established. Regardless, the lack of roost sites at the Verulam WWTW compared to the other study sites means that bats may incur higher energy expenditure to reach this feeding site, and the higher proportions of MUFA and PUFA in their adipose tissue will be beneficial both as a source of energy (Chalvardjian, 1964) and to optimise torpor usage (McGuire et al., 2013; Ruf and Arnold, 2008). The lower iBrAT mass of bats at both WWTW indicate a higher reliance on stored lipids to fuel energy demand.

Although a higher proportion of unsaturated fatty acids are beneficial for mitochondrial efficiency and energy saving, there is a trade-off between the benefits and cost of maintaining high levels of MUFA and n-6 and n-3 PUFA in tissues. Further, in mammals, high C16 and C18 desaturation indices (MUFA:SFA) are correlated with poor health outcomes (Jeyakumar et al., 2009; Sjögren et al., 2008; Warensjö et al., 2008). The higher degree of C18 desaturation, and the higher n-6 to n-3 PUFA ratio in the iBrAT of bats from Verulam WWTW may play a role in tissue degeneration (Frank et al., 1998) through ROS mediated HNE and superoxide production (Jastroch et al., 2010). HNE can affect tissues far removed from the tissue it was produced in (Ruf and Arnold, 2008), and together with rapid changes in body temperature associated with entry into and arousal from torpor (Willis, 2006) cardiac muscle have to be adequately protected against oxidative stress (Marber et al., 1995). One important protective measure is alteration in myocardial stress proteins and/or antioxidant enzymes (Yellon and Latchman, 1992). Bats at WWTW did not have higher lipid peroxidation levels in either pectoral or cardiac muscle compared with bats from reference sites. These bats either have the capacity to modulate mitochondrial efficiency when needed due to adequate PUFA levels in their diet or their antioxidant capacity is sufficient to protect these tissues against damage. This holds at least true for the non-breeding season in which we sampled bats for this study; lipid peroxidation levels may be higher during winter or the breeding season (Machlin and Bendich, 1987).

Literature on the effects of urbanisation and its associated pollution on iBrAT fatty acid profiles are scarce, yet previous studies highlighted changes in fatty acid profiles of deer sperm and duck livers as a result of mining-related metal exposure (Castellanos et al., 2010; Mateo et al., 2003). In addition, a recent study showed that artificial illumination may decrease BrAT activity in rodents (Kooijman et al., 2015), and tissue fatty acids have been shown to change as a result of selecting food with different fatty acid profiles (McGuire et al., 2013). WWTW feature all three potential stressors. Previous studies in our labs indicated that bats utilised the Verulam and Umbilo WWTW for feeding on the abundant midges and their renal metal levels were positively correlated with the water metal levels (Naidoo et al., 2013). Additionally, these bats were also susceptible to accruing micronuclei and DNA strand breaks in their blood cells (Naidoo et al., 2015), and renal lesions (Naidoo et al., 2016).

Our current results further highlight that the changes in BrAT fatty acid profiles in bats associated with WWTW may be in response to a combination of environmental stressors, although pectoral and cardiac muscle do not have high levels of lipid peroxides. While here we can only speculate on the effect of the changed BrAT fatty acid profiles on torpor and membrane functionality, we are currently assessing the baseline uncoupling protein-1 expression and the capacity to upregulate uncoupling protein-1 during arousal from torpor. Effects on basal metabolic rates and mitochondrial respiration rates will be especially insightful.

## MATERIALS AND METHODS

### Collection of samples

Pollutant-exposed *Neoromicia nana* bats were collected from two contaminated sites namely, Verulam WWTW (S29°38′38″; E31°03′49″) and Umbilo WWTW (S29°50′44″; E30°53′31″). Umdoni Park in Pennington (S30°23′39″; E30°.40′51″) and Buffelsdrift farm (S29°45′24.0″; E30°40′44.6″) were chosen as reference sites. Umdoni Park is a dense coastal forest that is situated further than 8 km from the nearest WWTW and Buffelsdrift farm is situated about 40 km west of Durban city, and 5 km from the nearest WWTW. Mist nets and harp traps were used to capture bats. Captured bats were sexed and only adults were selected for this study – the presence of cartilaginous epiphyseal plates aided in the determination of developmental stage (Anthony, 1988). Forearm length (to nearest 0.1 mm) and body mass (to the nearest 0.5 g) were measured using callipers and a Pesola^®^ (Baar, Switzerland) scale respectively. *N. nana* bats were identified using a taxonomic key (Monadjem et al., 2010) and other species that were captured were released. With the approval of the University of KwaZulu-Natal Animal Ethics Committee (Reference: 031/13/Animal), bats were euthanised humanely by decapitation and dissected for the collection of the iBrAT, heart and pectoral muscles. Tissues were snap frozen in liquid nitrogen, placed in labelled 1.5 ml microcentrifuge tubes and stored at −80°C for lipid, fatty acid and protein extraction.

### Fatty acid profile

Total lipids were extracted from 6-12 mg of iBrAT according to a modified Bligh and Dyer procedure (White et al., 1979) using a single-phase chloroform-methanol-aqueous buffer system in a ratio of 1:2:0.8 (v/v/v). Lipid fractionation was performed by silicic acid chromatography. The neutral lipid (NL) fraction was eluted with acetone and the phospholipid (PL) fraction with methanol. Fatty acid methyl esters (FAMEs) were prepared by mild alkaline methanolysis (Guckert et al., 1985). Samples were further derivatised with N,O-bis-trimethylsilyl-trifluoroacetamide (BSTFA) and trimethylchlorosilane (TMCS) to ensure silylation of compounds not readily methylated (Lindeque et al., 2013). FAMEs were analysed by capillary gas chromatography with flame ionisation detection on an Agilent (Santa Clara, CA, USA) 7890A gas chromatograph (split/splitless injector, 270°C) coupled to an Agilent 5975B mass-selective detector (electron energy 70 eV; ﬁlament current 220 μA, source temperature 230°C; electron multiplier voltage 2094 V, transfer line temperature 280°C). A 60 m SPB-1 fused silica column (0.25 mm ID, 0.25 µm ﬁlm thickness) was used with helium as the carrier gas at 1 ml min^−1^ constant flow. The oven temperature was programmed from 60°C (held for 2 min) to 150°C at 10°C per min, then from 150°C to 312°C at 3°C per min. Only phospholipid fatty acid profiles are discussed further in this paper, and fatty acids are expressed as a proportion of the total fatty acid levels as in McGuire et al. (2013) and Castellanos et al. (2010).

In addition to reporting the concentrations of FAMEs in the PL fraction of iBrAT lipids, we also calculated and reported the ratio between n-6 and n-3 PUFAs in the PL fraction and the C16 and C18 desaturation indices. The desaturation indices were calculated by the ratio of product to precursor fatty acids (C16:1 to C16:0 and C18:1 to C18:0 respectively) as in Jeyakumar et al. (2009).

### Total antioxidant capacity

Proteins were extracted from the heart and pectoral muscles using a method adapted from Van Heerden et al. (2004). Half of the heart and pectoral muscle samples were placed into fresh 2 ml microcentrifuge tubes together with 100 µl of whole cell extraction buffer (25% glycerol (v/v), 420 mmol l^−1^ NaCl, 1.5 mmol l^−1^ MgCl_2_, 0.2 mmol l^−1^ EDTA, 20 mmol l^−1^ Hepes, 0.5 mmol l^−1^ phenylmethylsulphonyl fluoride and 0.5 mmol l^−1^ dithiothreitol) and 1 µl HALT protease inhibitor (Thermo Fisher Scientific, Waltham, MA, USA). A stainless steel bead was added to each tube and the tissue homogenized for 3 min in a Tissuezyler LT (Qiagen^®^, Hilden, Germany). The stainless steel bead was removed using a magnet and samples were centrifuged at 13,500 rpm for 30 min. The supernatant was collected and placed in fresh 1.5 ml microcentrifuge tubes.

A Pierce™ BCA Protein Assay kit (Thermo Fisher Scientific, Waltham, MA, USA) was used for protein quantification against a BSA standard series (0, 25, 125, 250, 500, 750, 1000, 1500, 2000 µg protein ml^−1^). The absorbance was measured at 562 nm (Powerwave XS microplate spectrophotometer, BIO-TEK^®^, Winooski, Vermont, USA) using KC4 software.

A protocol by Griffin and Bhagooli (2004) of the ferric reducing antioxidant power (FRAP) assay was used to determine the antioxidant capacity of the heart and pectoral protein samples against a standard series of FeSO_2_.7H_2_O (0, 25, 50, 75, 100, 150, 200, 500, 1000 µmol l^−1^). Plate incubation time was extended to 20 min as suggested by Berker et al. (2007), and the absorbance was measured at 600 nm (Powerwave XS microplate spectrophotometer) using KC4 software.

### Lipid peroxidation

Total lipids were extracted from the heart and pectoral tissues using a modified Bligh and Dyer procedure (White et al., 1979), using a single-phase chloroform-methanol-aqueous buffer system in a ratio of (2:1:0.8 v/v/v).

The amount of lipid hydroperoxides in the pectoral and heart muscle tissues was determined using a PeroxiDetect™ kit (Sigma-Aldrich, St Louis, Missouri, USA). The protocol was based on the oxidation of Fe^2+^ to Fe^3+^ ions by hydrogen peroxides changing the colour of xylenol orange to red. A modification to the standard protocol was made by using tris (2-carboxyethyl) phosphine (TCEP) to measure the lipid-bound and aqueous hydroperoxides present in the tissue according to the method of Nourooz-Zadeh et al. (1995). A *tert*-Butyl hyderperoxide (t-BuOOH) standard series (0, 1, 2, 4, 8, 12, 16 nmol reaction volume^−1^) was used to determine absorbance at 560 nm (Powerwave XS microplate spectrophotometer).

### Statistical analysis

For statistical analysis, data collected from the single bat from Umdoni Forest were combined with data collected from the Buffelsdrift Farm bats, and we tested sample size for adequate study power of 80% (ClinCalc.com software, ClinCalc LLC). A Kolmogorov–Smirnov test was conducted on all data to test for normal distribution. We used two-way ANOVA followed by Tukey post-hoc tests to compare phospholipid fatty acid profiles among sites and between fatty acid classes or individual fatty acids. (GraphPad Prism 4, GraphPad Software, Inc., CA, USA). Because we found no significant differences in responses between sexes when comparing individual sites, Kruskal–Wallis statistics, followed by Dunn's multiple comparisons test were performed to test for differences in desaturation indices between sites, while one-way ANOVA and Tukey's HSD test were used to test for differences in n-6/n-3 ratio between sites.

To compare fatty acid composition of bats between polluted and unpolluted sites, we performed a non-metric multidimensional scaling (NMDS) analysis based on Bray-Curtis dissimilarities using the vegan package in R (v. 3.1.1 R Core Team, 2014). We also expressed individual fatty acids and fatty acid classes for each bat relative to the average of bats at the reference site in a heatmap using MultiExperiment Viewer software (v. 4.9, Dana-Farber Cancer Institute, MA, USA). A seven-fold increase in expression was set as a maximum, with all expression levels exceeding this level, indicated by the most intense colour.

To compare mean TAC and lipid peroxidase data among sites and between sex for those sites where we had enough males and females, two-way analysis of variance (ANOVA) followed by Tukey post-hoc tests, were used. Alternatively, one-way Kruskal–Wallis statistics, followed by Dunn's multiple comparisons test were used to test for significance between reference sites and individual WWTWs.
